# Enhancing Peptide Biomaterials for Biofabrication

**DOI:** 10.3390/polym13162590

**Published:** 2021-08-04

**Authors:** Kate Firipis, David R. Nisbet, Stephanie J. Franks, Robert M. I. Kapsa, Elena Pirogova, Richard J. Williams, Anita Quigley

**Affiliations:** 1 Biofab3D, Aikenhead Centre for Medical Discovery, St Vincent’s Hospital Melbourne, Fitzroy, VIC 3065, Australia; kate.firipis@student.rmit.edu.au (K.F.); rob.kapsa@rmit.edu.au (R.M.I.K.); elena.pirogova@rmit.edu.au (E.P.); 2 Biomedical and Electrical Engineering, School of Engineering, RMIT University, Melbourne, VIC 3000, Australia; 3 Laboratory of Advanced Biomaterials, The Australian National University, Acton, Canberra, ACT 2601, Australia; david.nisbet@anu.edu.au (D.R.N.); stephanie.franks@anu.edu.au (S.J.F.); 4 The Graeme Clark Institute, Faculty of Engineering and Information Technology, Melbourne, VIC 3000, Australia; 5 Faculty of Medicine, Dentistry and Health Services, The University of Melbourne, Melbourne, VIC 3000, Australia; 6 Department of Medicine, Melbourne University, St Vincent’s Hospital Melbourne, Fitzroy, VIC 3064, Australia; 7 Institute of Mental and Physical Health and Clinical Translation, School of Medicine, Deakin University, Waurn Ponds, VIC 3216, Australia

**Keywords:** biomaterials, biofabrication, bioinks, peptides

## Abstract

Biofabrication using well-matched cell/materials systems provides unprecedented opportunities for dealing with human health issues where disease or injury overtake the body’s native regenerative abilities. Such opportunities can be enhanced through the development of biomaterials with cues that appropriately influence embedded cells into forming functional tissues and organs. In this context, biomaterials’ reliance on rigid biofabrication techniques needs to support the incorporation of a hierarchical mimicry of local and bulk biological cues that mimic the key functional components of native extracellular matrix. Advances in synthetic self-assembling peptide biomaterials promise to produce reproducible mimics of tissue-specific structures and may go some way in overcoming batch inconsistency issues of naturally sourced materials. Recent work in this area has demonstrated biofabrication with self-assembling peptide biomaterials with unique biofabrication technologies to support structural fidelity upon 3D patterning. The use of synthetic self-assembling peptide biomaterials is a growing field that has demonstrated applicability in dermal, intestinal, muscle, cancer and stem cell tissue engineering.

## 1. Introduction

Evolution has equipped the body with an incredible capacity to heal injured and diseased tissues [1,2]. However, when the volume and complexity of damage overcomes endogenous repair mechanisms, healthy tissue regeneration often fails [3,4]. There is a clinical need for improved tissue replacement techniques, as severe tissue loss leads to functional limitations and negatively impacts quality of life [5,6,7]. Biofabrication is recognised as an emerging pathway for the effective regeneration of diseased or injured human tissues [8,9], relying on both technological and material scientific advances to develop appropriate scaffolds and bioinks. The development of bioinks and biofabrication strategies aims to support the repair of tissues or, more ambitiously, provide life-saving lab-made functional organs or tissues for implantation.

Organs in the body are organised in a three-dimensional (3D) hierarchical manner [10,11,12]. Biofabricated constructs should aim to mimic these 3D cellular interactions that ultimately affect functional activity [13,14,15,16,17,18,19,20,21]. The field of biofabrication aims to replicate the native hierarchical tissue and organ structure by placing biomaterials and cells precisely into a 3D space, creating living constructs [22,23,24,25,26,27,28,29,30]. These 3D models of native organ structures can be captured from magnetic resonance imaging (MRI), computed tomography (CT) or designed in computer-aided design (CAD) programs and translated to control biofabrication patterning [9]. The accuracy of the fabricated design depends on the resolution of biofabrication technologies and amenable bioinks. Current biofabrication technologies include inkjet printing [31], laser-assisted printing [32,33], extrusion printing [34,35], molding [36] and freeform fabrication [37]. As well as advances in biofabrication [9], development of more sophisticated bioreactors [38], vascularisation [39] and innervation [40] strategies, and further progress into enhanced bioinks is required [41]. Biofabrication poses significant challenges for translating existing biomaterials into bioinks [42]. For example, bioinks for extrusion bioprinting require material properties, such as a high viscosity, shear recovery and rapid stabilisation [43,44]. In tandem, bioinks should present extracellular matrix (ECM) mimetic cues to promote the desired cellular behaviours [13,45,46,47]. There is a paucity of bioinks that meet all of these criteria. The lack of bioinks that are amenable to biofabrication, preserve cellular integrity during the bioprinting process and present controlled ECM-mimetic cues—combined with the need to address issues surrounding vascularisation and innervation—is limiting the research field.

The native ECM is a highly hydrated self-assembling hierarchical scaffold, comprised of tissue-specific molecules, including structural and functional proteins and polysaccharides (e.g., collagen, elastin, fibronectin, laminin and glycosaminoglycans) of different sizes and shapes as well as soluble signalling molecules [48,49]. The ECM scaffold provides tissue-specific structural and functional properties [50], established to provide primary points of interaction that drive cellular migration, differentiation and proliferation—essential behaviours for tissue engineering [13,45,46,47]. These cell-scaffold interactions are thought to be a combination of signals from the scaffold’s mechanical properties [51,52,53,54], structure [55,56], and bioactivity [57,58]. Together, these tissue-specific mechanical, structural and bioactive signals make up an ‘extracellular niche’ that can influence cell behaviour [50]. Significant progress has been made to translate the knowledge of native tissues’ structural and functional properties to lab-made scaffolds [59,60,61]. Controlled scaffold cues have been shown to influence and drive cell behaviour towards functioning engineered tissues [25,26,27,28,29,30]. This demonstrates the importance of controllable and engineered extracellular cues; without access to reproducible materials, it is challenging to fully control cell–scaffold interactions and manufacture quality-controlled matrices for tissue and organ engineering.

Historically, lab-made scaffolds have been synthesised from modified proteins or long-chain polysaccharides [62,63,64]. However, protein materials sourced from animals suffer from batch-to-batch inconsistency and xenogeneic protein transfer issues, limiting translation to clinical settings [65,66,67]. Inconsistencies in cell-scaffold interactions and biofabrication outcomes undermine the use of natural protein and polysaccharide materials for tissue engineering. While methods are being developed to screen material batches for variation outside tolerances [68,69], advances in synthetic material design have created consistent but somewhat underutilised materials in the biofabrication field [70].

The regulatory approval of biofabricated organs remains of utmost importance for clinical translation. Appropriate guidelines for approval remains an ongoing discussion, however is likely to include the reporting of manufacturing tolerances [71]. Approval is a lengthy and resource-heavy task that can be alleviated by using innately reproducible materials, reducing variability in the product. To design tissue-specific ECM-niches, the ideal biomaterial for biofabrication is engineered for cellular outcomes, with mechanical, structural and bioactive properties presented in a controlled manner [45,72,73]. Advances in synthetic biomaterial design have allowed researchers to design bespoke synthetic materials, such as bicyclic-RGD-modified polyethylene glycol, (PEG) with the presentation of ECM-niche cues that are integrin selective [74]. Although significant progress has been made using highly reproducible materials such as PEG [75], another synthetic material class, synthetic self-assembling peptide hydrogels, have already demonstrated several improved beneficial properties: bioresorbable, biodegradable, and biocompatible [76,77,78]. The following reviews on synthetic biomaterials provide a broader overview of the field [70,79].

**Figure 1:**
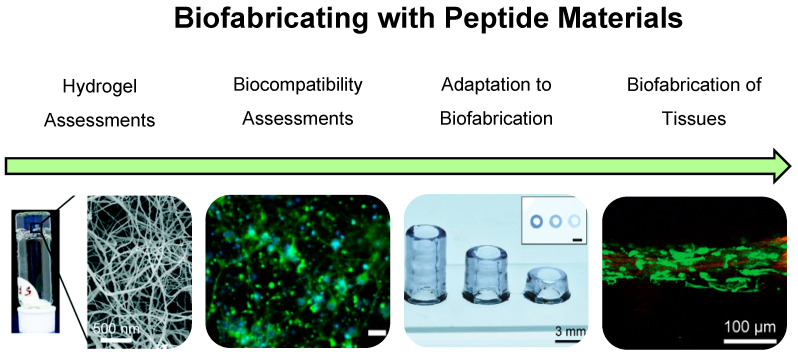
The road to biofabrication with peptide-based materials involves the discovery of self-assembling peptides that form hydrogels with ECM-mimetic properties. The materials undertake assessment to determine biocompatibility with a range of cell types as seen here with cortical neurons. Furthermore, the adaptation of hydrogel mechanical properties and the development of unique biofabrication strategies for peptide–biomaterials are developed, enabling high shape fidelity and the precise deposition of biocompatible peptide–biomaterials. The emergence of cell laden peptide-biomaterials which can be biofabricated demonstrates the successful translation of peptide materials to the biofabrication of tissues. Images adapted with permission from Rauf et al. 2021 CC BY-NC 3.0 published by RCS [80], Susapto et al. 2021 Copyright ACS [81] and Sather et al. 2021 Copyright Wiley-VCH [82].

Progress into biomaterial molecular modelling and design principles may improve the clinical translation of materials by predicting outcomes without significant labour-intensive bench time. Molecular modelling [80,81,82,83,84], design principles [85,86,87,88,89,90,91] and predictive gelation models [90] of synthetic peptide materials are being increasingly reported, indicating a future ramp-up of high-throughput peptide biomaterial discovery. Synthetic self-assembling peptide (SAP) hydrogels are peptide sequences that self-assemble via supramolecular interactions to spontaneously immobilise fluid, creating a highly hydrated scaffold [92]. These SAP materials have been designed to mimic the native ECM structure, function and self-assembly mechanisms [93,94,95]. Synthetic peptide materials give rise to complex biomimetic structures with bioactivity, resulting in controlled cell-scaffold interactions [96,97]. Furthermore, recent reports have demonstrated the translation of synthetic SAP biomaterials into bioinks [80,81,82,98,99,100,101,102,103,104,105]. This demonstrates the potential of synthetic SAP design for biofabrication of organs and tissues, and ultimately clinical translation (Figure 1).

In this review, we provide a commentary on the recent progress of adapting synthetic peptide materials as effective biomaterials and the mechanistic approaches that have been taken to ensure their development in the biofabrication landscape. This review highlights synthetic peptide materials that recapitulate key features of the ECM, paving the way to the biofabrication of tissue-engineered organs and future clinical translation.

## 2. A Brief History of Peptide Hydrogels as Biomaterials

**Figure 2:**
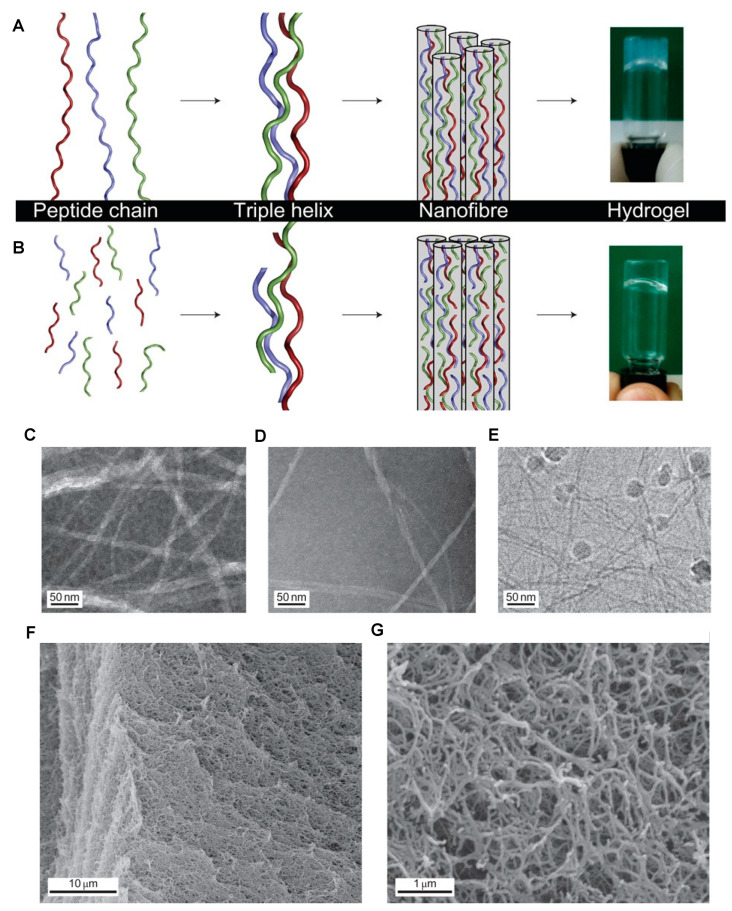
(**A**) Type I collagen assembly in which the peptide chains (shown in red, blue and green), consisting of ~1000 amino acids, form triple helices ~100 nm in length and the blunt-ended nanofibres (shown in grey) assemble via the staggered lateral packing of the triple helices. The hydrogel pictured represents rat-tail collagen. (**B**) Self-assembly of collagen mimetic peptides, in which the peptides consist of 36 amino acids (shown in red, blue and green), form a triple helix staggered with a length of 10 nm and the nanofibres (shown in grey), and result from triple helical elongation, as well as from lateral packing. The hydrogel depicted is the synthetic peptide (Pro-Lys-Gly)_4_(Pro-Hyp-Gly)_4_(Asp-Hyp-Gly)_4_. (**C**–**E**) Transmission electron microscopy (TEM) images of collagen-like nanofibres taken at ×40,000. (**F**,**G**) Scanning electron microscopy (SEM) images of critical-point dried hydrogel with a peptide concentration of 1.0% by weight that shows the interconnected fibrous structure responsible for the gel forming properties at ×3100 (**F**) and ×30,000 (**G**). Adapted with permission from O’Leary et al. 2011 Copyright Nature publishing [114].

Proteins and peptides in the body serve as the foundation for structures such as the cellular cytoskeleton, ECM components such as collagen, and the cell-membrane integrins that mediate molecular recognition between cells and the ECM [106,107]. The serendipitous discovery in 1995 that the synthetic peptide Fmoc-LD (containing amino acids leucine and aspartic acid and capped with a Fmoc-group) self-assembled into nanofibres and further immobilised surrounding fluid to form a hydrogel network [108], has spurred the development of a range of synthetic peptide hydrogel systems. Several of these peptide systems, including Fmoc-FF [109], have been found to be cytocompatible and able to support the culture of a range of cell types. Cytocompatibility, and also biocompatibility, of peptide hydrogels was to be expected as peptides and the bonds between them are known to cells and to the body. As seen in the ECM, collagen assembles into fibres (Figure 2) and contains bioactive sites (such as RGD). Adapting to the designable nature of peptide sequences, researchers have varied peptide sequences towards the presentation of biomimetic structures and bioactive sites (e.g., RGD), resulting in the formulation of designed biomaterials. [96,110]

### 2.1. Structural Protein Mimics

The native ECM contains proteins such as collagen and elastin, which provide structural and functional cues to resident cells. However, many sources of natural protein biomaterials are animal-derived [65,66,67]. Synthetic peptide biomaterials have made significant progress in mimicking the structural and functional cues of native proteins and may provide an alternative to many naturally derived proteins [111,112].

Collagen is a major component of ECM architecture and plays an integral role in cell attachment. Collagen-mimetic synthetic peptide hydrogels have demonstrated features of native collagen such as α-helical structure [87,113,114,115,116,117,118,119,120,121] (Figure 2) and degradation by collagenase enzymes [114]. Further, collagen-mimetic hydrogels have been designed to represent more specified ECM-niches by the inclusion of specific binding motifs. Bioactive modification (RGDS motif) [122] and the ability to support several niche cell types, including neural PC12 [122], 3T3 fibroblasts [122], and murine embryonic neural stem cells [123], have been reported. Collagen-mimetic hydrogels have been shown to work as a hemostat [115] for drug-release [124], and have demonstrated a pro-healing macrophage profile after 28 days post-implantation [125]. These findings reveal the potential of collagen-mimetic synthetic SAP biomaterials for diverse tissue applications.

Native elastin-mimetic peptides also have the potential to contribute to the mechanical properties of synthetic hydrogels. Native elastin lends elasticity to the ECM via insoluble elastin fibres, comprised of β-turns. To synthetically mimic elastin, elastin-like polypeptides (ELPs) have been developed [126]. ELP materials have proven to be biocompatible, biodegradable, non-immunogenic and can be produced with a high yield [127]. Moreover, these materials maintain the growth of several cell types and tissue explants, including chondrocytes [128], dorsal root ganglia [129], cochlea corti [130], and embryonic stem-cell-derived cardiomyocytes [131]. Of particular interest is a study reported by Chang et al. in 2015, where alternating peptide sequences of elastin-like and bioactive fibronectin-like (RGD) motifs formed both a bioactive and elastic hydrogel [130]. This demonstrated the potential of ELPs to maintain diverse cell-types in culture, to modify biomaterials to present ECM-niche properties and the versatility of synthetic SAPs to mimic natural proteins.

### 2.2. Modified Peptide Materials

Synthetic peptide materials are compatible with existing material modification techniques aimed at improving the tissue-specificity of the final material [96,132,133,134]. Synthetic peptide materials have demonstrated the ability to be mechanically, structurally, and bioactively modified to mimic tissue-specific scaffold features [111,135,136]. The ability to manipulate the physical and mechanical properties of synthetic SAP hydrogels by altering peptide sequences or assembly conditions is important for versatility in bioengineering applications. Approaches to vary the mechanical properties of synthetic protein networks relies on the formation of covalent crosslinks, and increases in polymer concentration or control of assembly [110,134,137,138,139,140]. Hydrogelation of SAPs is crucial to create a mimic of the highly hydrated and bioactive ECM scaffold. Significant progress has been achieved in developing a range of gelating synthetic SAPs including MAX-peptide [141,142,143,144,145], F-peptide [146,147,148,149], Y9-peptide [150,151], RAD16-peptide [78,152,153,154,155,156,157], EAK-peptide [158,159], Fmoc-peptide [96,97,110,160,161,162,163,164,165] and peptide amphiphiles (PA) [166,167,168,169].

MAX1 and MAX8 are examples of β-hairpin SAPs that are biocompatible and have demonstrated support of osteoblasts [170]. In comparison, F-peptide, RAD16 and EAK16 form β-sheet containing hydrogels, which have also demonstrated biocompatibility. F-peptide has been modified for the presentation of ECM-niche bioactive RGD motifs [148,149]. This method allows for the independent control of stiffness and density of RGD motifs, and enables the growth of human umbilical-vein endothelial cells (HUVECS) and human mesenchymal stem cells (hMSCs) [148,149]. RAD16, also known commercially as Puramatrix, has also demonstrated ECM-niche design ability [157]. By introducing fibroin peptide sequences, the mechanical stiffness of the resulting hydrogels could be increased [157]. Further, RAD16 has shown the ability to deliver osteogenic bioactives, significantly promoting proliferation and the cellular expression of osteogenic differentiation markers [156]. EAK16-peptide, similar to F- and RAD16- peptides, was able to be modified for an ECM-niche design. Conjugation with bioactive motifs (representing fibronectin, laminin and vitronectin) allowed the attachment of neural cells and neurite development [158]. This demonstrates the broad range of peptide biomaterials that have been developed. The biomaterials are shown to be highly hydrated, bioactive scaffolds with the ability to be designed for ECM-niches to support a wide range of cell types.

**Figure 3:**
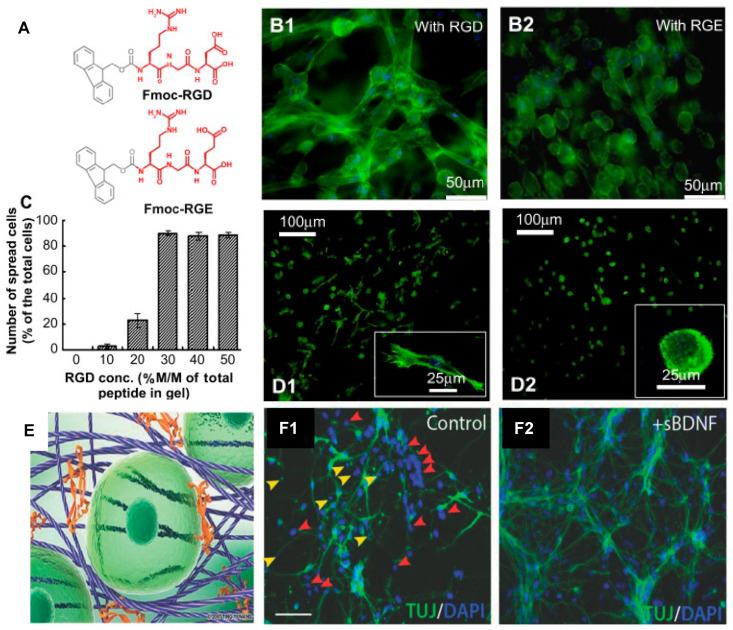
**Biocompatibility of self-assembling peptide hydrogels**. The Fmoc-FF/RGD hydrogel promotes cell adhesion with subsequent cell spreading and proliferation. (**A**) The structures of the two chemical analogs: Fmoc-RGD and Fmoc-RGE. (**B**) Cell adhesion and morphology in the Fmoc-FF/RGD and Fmoc-FF/RGE hydrogels: (**B1**) human adult dermal fibroblasts (HDFa) are well-spread in the Fmoc-FF/RGD hydrogels and form a three-dimensional cell network 48 h post culture; (**B2**) HDFa in the Fmoc-FF/RGE hydrogels maintains a round morphology after 48 h. (**C**) The Fmoc-RGD concentration also influenced cell spreading; in the hydrogels with 30–50% Fmoc-RGD incorporated, adequate cell spreading occurs with over 90% spread cells. (**D**) Integrin blocking experiments proved direct interaction of the cells with RGD after 20 h: (**D1**) Cells with unblocked α5β1 integrins were able to spread and directly attach to the RGD sites on the nanofibres. (**D2**) Cells with blocked α5β1 integrins were unable to attach to the RGD sites and remained rounded. (**E**) Fmoc-DDIKVAV nanofibres interact with one another, forming a nanofibrous network, into which proteins such as the brain-derived neurotrophic factor (BDNF) (orange) can be shear-encapsulated to sustain delivery, thereby providing structural and chemical support for cells (green). (**F1**) Representative cortical primary cultures illustrating total DAPI labelled cells and proportionate TUJ+ neurons under control conditions and following (**F2**) soluble brain-derived neurotrophic factor (sBDNF) treatment. Note the increase in pyknotic nuclei (yellow arrows), as well as DAPI+ cells failing to adopt a TUJ+ neuronal fate (red arrows) in the absence of BDNF. Scale bar 100 µm. (**A**–**D**) Adapted with permission from Zhou et al. 2009 Copyright Elsevier [110]. (**E**,**F**) Adapted with permission from Nisbet et al. 2018 Copyright Wiley-VCH [171].

The Y9-peptide, Fmoc-peptides and peptide amphiphiles demonstrate a range of β-sheet, random coil and α-helical structures in their nanofibrous materials along with amenability to tune bioactive motifs. In particular, Y9 has been modified with the RGDS motif conferring greater bioactivity to the hydrogels [150,151]. Fmoc-peptides have also been developed to include a range of bioactive sequences for the improved mimicry of the bioactive ECM-niche. Motifs including RGD and RGDS [96,110,161,162,163,165], IKVAV and YIGSR [97,163,164,165,171], and GFFGER [165] have been incorporated into Fmoc-peptides to mimic the key motifs in fibronectin, laminin and collagen, respectively, and promote cell attachment (Figure 3). Peptide amphiphiles have also been modified with the laminin or fibronectin motifs, IKVAV [167] or RGD [169], respectively. Furthermore, these SAPs have demonstrated ability to support a range of cell-types in culture. The modified Y9-peptide supported PC12 and fibroblast cell growth [150,151]. Fmoc-peptides have demonstrated the support of human adult dermal fibroblasts [110], human mammary fibroblasts [96], L929 fibroblasts [165], neural cells [163,171], and C2C12 mouse myoblasts [162], (Figure 3). Peptide amphiphiles have shown support and induction of neuroectodermal lineage from MSCs [167].

To demonstrate the range of mechanical properties achievable with the bioactive Fmoc-FRGDF system, Li et al. used phosphate-buffered saline (PBS) at varied ionic strengths (0.25–0.75 M) and controlled the time to pH equilibrium to create materials with mechanical properties in the range of 10 Pa to 11 kPa and with the ECM-motif, RGD [134]. Li et al. discussed that the mechanical control was facilitated by the assembled network’s underlying organisation. An increase in disordered and entangled structures was observed with increasing PBS concentrations. A correlation between increased PBS concentrations and mechanical properties of created networks was also observed. This indicated that the hydrogel’s mechanical increase was a result of underlying disordered and entangled structures in the fibrous networks [134].

SAPs can also be blended to form co-assembled hydrogels [110,139,140,162]. To achieve this, peptides are combined before assembly, allowing for the formation of complex networks consisting of fibrils of mixed sequence. Co-assembled networks of Fmoc-YIGSR and Fmoc-IKVAV, two laminin-mimetic peptides, enhanced the network mechanics of hydrogels from 674 Pa (Fmoc-YIGSR) and 267 Pa (Fmoc-IKVAV) to 937 Pa [140]. The increase in mechanical properties was attributed to the interactions of hydrophobic and hydrophilic peptides and increased hydrogen bonding. This method also demonstrated that by varying the peptide ratios, the mechanics could be varied further. In elastin-like and resilin-like polypeptides, lysine residues (K amino acid) could be added to create crosslinking potential for mechanical control [172,173]. Materials prepared by this method, depending on the percentage of crosslinks formed and the number of K-edited domains, facilitated a mechanical modification between 1 kPa and 1 MPa. These reports demonstrate that the variation of SAP hydrogel properties, by altering assembly conditions, allows for a wide range of mechanical properties, inclusive of the range of mechanical properties seen in many tissues of the body. This is achieved by altering covalent crosslinking, concentration changes, ionic or pH changes, and coassembly with other SAPs.

The native ECM includes various structural and functional proteins and polysaccharides that self-assemble into a supramolecular network, the complexity of which is yet to be fully recapitulated in lab-made scaffolds. Reports describing the combination of SAPs with other macromolecules have demonstrated that hybrid networks present beneficial properties and facilitate the mechanical tuning of the hydrogels for improved tissue-mimicry. Fmoc-peptides combined with macromolecules agarose [133], versican [111] and/or fucoidan [111,135,136], have demonstrated the ability to vary the scaffolds’ mechanical properties [111,133,135,136], as well as demonstrating anti-cancer [135] and anti-inflammatory [95,111,136] properties.

### 2.3. In Vivo Applications of Peptide Materials

SAPs have also been used for in vivo cell and drug delivery. Fmoc-FRGDF, Fmoc-DIKVAV and Fmoc-DYIGSRF were used to deliver cortical progenitors into the brain of C57BL/6 mice, demonstrating the improved delivery and viability of cortical neural progenitor cells and a limited foreign body response to the material [163]. In vivo assessment of Fmoc-DDIKVAV in a mouse stroke model demonstrated that the hydrogel’s structural and bioactive functional support promoted stem cell integration (human progenitor stem-cell-derived cortical neurons) and differentiation, reduced tissue atrophy and improved the recovery of motor function over nine months [164]. Furthermore, Fmoc-peptides and peptide-hybrid hydrogels showed biocompatibility and supported tissue regeneration when implanted into the brains of mice subjected to acute traumatic brain injury [136].

Peptide amphiphiles have also demonstrated in vivo applications, such as a drug carrier for an atherosclerotic plaque-reducing drug [168]. Additionally, when modified with RGD, PA materials can promote wound healing after burns [169]. Further, in vivo applications of the SAP RAD16 include efficacy as a haemostat [153], facilitating tissue reconstruction after central nervous system (CNS) injury [78] or bone defects [154], and providing anti-cancer microenvironmental cues [155]. This demonstrates that SAP and hybrid materials can support tissue regeneration and influence tissue response by mimicking elements of the native ECM. In future, clinical applications of Fmoc-peptides, peptide amphiphiles and RAD16 may include cell delivery for stroke recovery, drug delivery, wound regeneration, haemostat, CNS repair, bone repair and anti-cancer treatments.

## 3. Adapting Peptide Materials as Bioinks

Biofabrication requires materials with properties that can both support cellular survival and growth and retain a designed 3D structure to result in a functioning living scaffold. Several studies have investigated the role of material properties that predict suitability for bioprinting, such as viscosity [174,175,176,177,178], shear-thinning [177,178,179,180,181,182,183], loss tangent [184], and, more recently, yield stress [184,185]. However, in the translation of synthetic peptide materials to bioinks, these key predictors are often under-reported and seemingly inconsistent. In reported studies of SAP bioinks, measures of printability such as viscosity [80,81,82,104], loss tangent [80,81,102], shear-thinning [81,82,103], achievable height [81,82,99,103] and filament assessments [81,82,103], were briefly discussed. This demonstrates the adoption of printability measures into the SAP bioink field; however, the lack of standard printability outcomes and the inconsistency in relationships of printability and predictors such as viscosity [81,82] limit the understanding of key material properties for future development.

There is a significant lack of knowledge in the mechanisms and design principles that create a bioprintable peptide material. In attempts to address this, Sather et al. investigated the relationship between peptide assembly and viscosity [82]. The authors reported that the bundling of fibres increased the bulk viscosity of the bioink, which improved printability as seen by the maintenance of deposited shape. Specifically, this bundling was accounted to hydrophobic residues on the nanofibre surface. Furthermore, post-printing, the authors reported that the variation of salt valency (for ionic crosslinking) affected both filament width and stiffness of the bioink. The authors also reported that transition of the SAP assembly from fibre to spherical micelle reduced printability. This contradicts the findings of Nolan et al., who reported that spherical domains improved printability [103]. However, differences in data reporting between the studies (i.e., Sather et al. directly related printability to increased viscosity, compared to Nolan et al., who did not report viscosity), clouded the relationship of assembly and printability. These studies indicated the need for minimum data reporting of bioink predictors across studies of SAP materials to make future comparisons. These reports all used custom-made biofabrication setups, indicating the difficulty of translating SAP biomaterials to current biofabrication technologies.

Traditional biofabrication techniques have incompatibilities with SAPs, currently limiting the use of this material class. Many techniques for biofabrication exist, including inkjet printing [31], laser-assisted printing [32,33], extrusion printing [34,35], molding [36] and freeform fabrication [37]. Compared to traditional bioinks, SAP materials have different gelation conditions, often involving salt solutions (Table 1). This has required the development of novel fabrication setups to enhance gelation. Bioprinting techniques amenable to SAP bioinks include droplet printing [98,99,100,101], or the generation of droplets which are then extruded [100,101], extrusion printing [80,81,102,103,104], and the customisation of extrusion printing setups, such as coaxial nozzles to mix salt solutions [80,81], printing onto salt-covered substrates [82], or removing excess fluid with a vacuum print-bed [105]. This demonstrates that the unique properties of SAP materials can be exploited for bioprinting. However, it is evident that further development in both SAP bioinks and bioprinting methods will be needed to facilitate the shape fidelities of printed constructs.

Hybrid materials can beneficially combine properties to promote printability. An alternative design principle for bioprintable peptide materials is the combination of other molecules with peptides [34,186,187,188,189,190] (Table 1). These bioinks support printability by complementary interactions, promoting viscosity [34], robustness [186], or mechanical recovery [187,188,190].

**Table 1:** Summary of SAP bioink materials, bioprinting techniques, printability outcomes, assembly, mechanics and cell types that were assessed.

Hybrid	Material	Bioprinting Technique	Printability Outcome/s	Assembly	Mechanics	Cell Type/s	Ref
No	LIVAGK ILVAGK and derivatives	Microfluidic flow focusing system (nanoparticles, custom two-inlet nozzle)	Not reported	Beta turn	40 kPa	Human H1 ESCs, hMSCs HUVECs, Fibroblasts Keratinocytes & Caco2	[98]
No	IVFK IVZK	Coaxial microfluidic nozzle (converted Dobot Magician printer)	V, LT	Beta turn	6–100 kPa	Human dermal fibroblasts hMSCs	[80]
No	IIFK IIZK IZZK	Extrusion Printing (Dual-Coaxial nozzle)	V, LT, ST, AH, FA	Beta-turns and beta-sheet	1–108 kPa 6–271 kPa 4–315 kPa	Human dermal fibroblasts hMSCs	[81]
No	E3 K3	Direct ink writing (extrusion based) onto salt-coated substrates	V, ST, AH, FA	Beta-sheet	0.03–12 kPa 0.01–1.5 kPa	C2C12	[82]
No	PeptiGelDesign.Ltd (Manchester BioGel)	Extrusion Printing (3D discovery, regenHU)	LT	Not reported	10 kPa (Alpha1) 1 kPa (AlphaProB)	Mammary epithelial cells	[102]
No	Fmoc-FF	Extrusion Printing (RepRap)	ST, AH, FA	Not reported	1 kPa	None reported	[103]
No	Fmoc-YD + Fmoc-YK	Droplet Printing (CellJet)	AH	Anti-parallel beta sheet	4 to 62 kPa	Human hepatoma spheroids	[99]
Yes	Nap-FFK-acrylic acid + PEGMA	Extrusion Printing (Nano-Plotter NP 2.1, GeSiM)	ST, FA, SR	Not reported	1 kPa	NIH-3T3 cells	[187]
Yes	Thiolated-gelatin + PA	Extrusion Printing (EnvisionTEC, 3D-Bioplotter)	ST, FA	Not reported	1 kPa	SV40 immortalised mouse cholangiocytes	[188]
Yes	PA + fibronectin, collagen, keratin, elastin-like proteins or hyaluronic acid	Inkjet Printing (custom, into supporting bath of one component)	V	Beta-sheet	0.5–0.9 kPa	NIH-3T3 adipose derived stem cells	[189]
Yes	poly(benzyl-L-glutamate)-*b*-oligo(L-valine)	Extrusion Printing (custom)	SR	Not reported	1.5 kPa	BaIb/3T3 fibroblasts	[190]
Yes	RAD16-I + methylcellulose	Extrusion Printing (3D Discovery Printer)	V, FA	Beta-sheet	10 kPa	Human MSC derived from adipose tissue & Rat MSC	[34]

V: Viscosity, LT: Loss Tangent, ST: Shear-Thinning, AH: Achievable Height, FA: Filament Assessments, SR: Shear Recovery.

## 4. Bioprinting of Tissues and Tissue Models with Self-Assembling Peptide Bioinks

### 4.1. In vitro Tissue Engineering with SAPs

SAP bioinks are currently in development, and already in use, for a wide range of in vitro tissue reconstruction and modelling applications. Progress has been made in the biofabrication of skin, organ structures, muscle tissue, and the modelling of cancer. However, many of these systems remain in the very early stages, and a number of further advances are required before SAP bioinks can be adopted more broadly.

The development of SAP bioinks for dermal bioprinting requires the capability to support resident cell types in the epidermal, dermal, and subcutaneous skin layers. Progress into the development of skin models using SAP bioinks has demonstrated cytocompatibility with fibroblasts [80,81,98]. The droplet printing of SAP gels (Table 1) demonstrated efficacy as a model of skin [98]. In a study by Loo et al., HUVECS and fibroblasts peptide droplets were deposited side by side, and keratinocytes were then seeded onto the apical surface to make a two-layered skin model [98].

**Figure 4:**
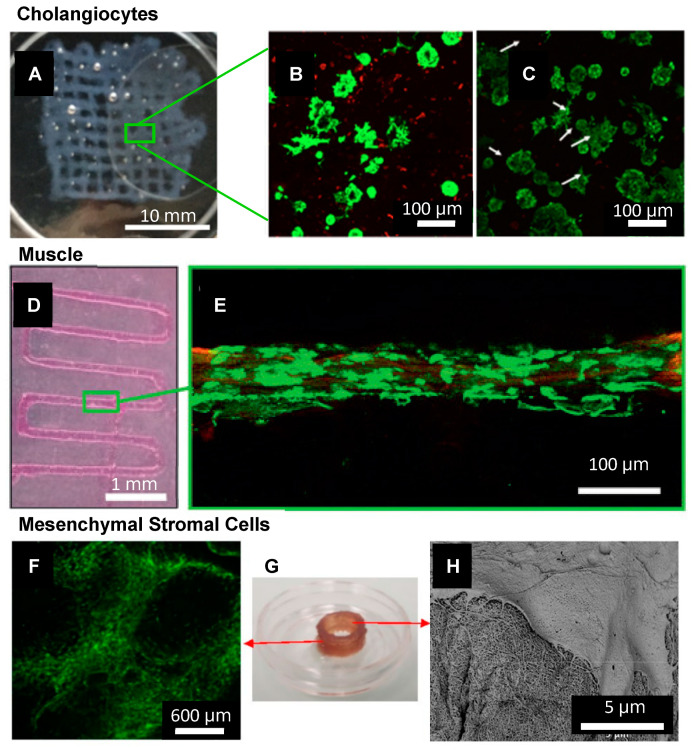
SAP bioinks have been used for the biofabrication of several cell/tissue types including (**A**–**C**) **Cholangiocytes**. (**A**) 3D bioprinting IKVAV-ink via extrusion through a 250 μm tip into a 15 mm × 15 mm grid, treated by secondary crosslinking solution. Scale bar is 10 mm. (**B**) Live/Dead stain of cholangiocytes in IKVAV-ink for 7, and (**C**) 14 days. Scale bars are 100 μm. Adapted with permission from Yan et al. 2018 Copyright IOPScience [188] (**D**,**E**) **Muscle**. (**D**) 3D bioprinting of PA-bioink on a CaCl_2_-coated glass coverslip using a 200 µm nozzle. Scale bar is 1 mm. (**E**) Confocal image of myoblast cells encapsulated in a filament after seven days in culture, stained with Calcein-AM (green) showing live cells aligned along the fibre axis. Scale bar is 100 μm. Adapted with permission from Sather et al. 2021 Copyright Wiley-VCH [82]. (**F**–**H**) **MSCs** (**F**) Long-term (30 days) cell viability of hBM-MSCs post-printing of a 1 cm cylindrical construct using IZZK peptide. Scale bar 600 μm. (**G**) Printed construct after 30 days (**H**) SEM of printed hBM-MSCs after ten days of culture showing an interaction between the cell’s filopodia and the matrix. Scale bar 5 μm. Adapted with permission from Susapto et al. 2021 Copyright ACS [81].

A SAP bioink has also been used to develop an intestinal epithelial model, where Caco2 cells formed fully confluent sheets with the anatomical features of intestinal tight junctions and developing microvilli [98]. Furthermore, a study of bile duct cells (cholangiocytes) in a SAP hybrid bioink indicated the formation and growth of cysts that budded and formed branching tubular structures after one week [188] (Figure 4A–C). These engineered intestinal structures could potentially be used for disease modelling as well as drug testing.

Biofabrication is also a promising technique for the development of structurally and functionally appropriate muscle for implantation or in vitro testing. Only a small number of articles have reported on self-assembling bioinks for the culture of muscle cells [82,104]. A recent study by Sather et al. presented a SAP bioink with an aligned nanofibrous topography that supported the alignment of C2C12 muscle cells [82] (Figure 4D,E).

The development of cancer models is an important step in testing for anti-cancer compounds. A recent review indicated that SAPs were promising for the local delivery of anti-cancer compounds [94]. A study of a SAP bioink demonstrated that Fmoc-YD/Fmoc-YK was compatible with the formation of human hepatoma cells HepaRG spheroids [99]. The formation of spheroids is a key step in cancer research due to the three-dimensional cues that impact cellular behaviour in native cancer structures. This demonstrated the potential of SAP bioinks to develop cancer models for the testing of anti-cancer compounds in the future.

### 4.2. SAP Support of Stem Cell Proliferation and Differentiation

A valuable avenue of research is the development of bioinks that can support stem cells. Stem cells are extensively used in tissue engineering applications to facilitate the remodelling of tissues. Stem cells have been used clinically in macular degeneration [191,192] and myocardial infarction [193], where data show they are safe and well-tolerated [191,193,194]. In the field of SAP and hybrid bioinks, stem cells have not been widely reported. However, reports have described the support of stem cells [80,81,98,189] and the ability to induce multipotent cells to specific lineages [81,98]. Peptide droplets were used to culture human H1 embryonic stem cells, which demonstrated the expression of pluripotent nuclear transcription factors and surface markers [98]. The same material was used to culture human mesenchymal stem cells (hMSCs), which demonstrated cell elongation and alignment [98]. Cells were induced to adipogenic lineage and demonstrated features of adipogenesis [98]. Similarly, an SAP hybrid ink supported the viability of hMSCs and the differentiation of rat MSCs into adipogenic linage [34]. Another study demonstrated that within an SAP bioink, printed human bone marrow mesenchymal stem cells could be sustained in culture for up to 30 days [81] (Figure 4F–H). Compared to Matrigel, cells in the peptide bioink demonstrated improved viability and the sustained potential for osteogenic, adipogenic and chondrogenic differentiation [81]. These reports demonstrated that SAP bioinks could support the growth and differentiation of various stem cells. However, significant work remains to reflect the complexity of the ECM-niche in SAPs which can be biofabricated, particularly for the control of stem cell behaviour.

## 5. Conclusions and Future Outlook

The field of biofabrication is compelling because of its potential to provide solutions to many human ailments and significantly improve quality of life. Biofabrication techniques have responded to the knowledge of 3D cell-scaffold interactions, generating novel solutions to fabricate hierarchical biomimetic structures. The future of biofabrication lies in the development of effective bioinks that not only provide niche cell-scaffold interactions, but create a hierarchical, truly mimetic lab-made tissue or organ. However, progress still needs to be made in the development and refinement of synthetic bioinks. SAP-based hydrogels offer a unique opportunity to tailor bioinks for biofabrication from the molecular level. SAP bioinks facilitate the building of multiscale cues ranging from bioactive motif–cell interactions to the structural nanofibrous topographies and the bulk mechanical properties of ECM-niches.

The innovation of synthetic SAP biomaterials is important not only in terms of creating native protein-mimetic biomaterials, but also in terms of the major impact on the preconceived ideas of biomaterial design. In the years following the innovation of synthetic SAP biomaterials, the lack of SAP use in biomedical applications gave way to an increasing body of work which studied in vivo regenerative medicine, 3D cell culture and biofabrication, seen most recently. Progress has been made to adapt synthetic SAPs to bioinks for dermal, muscle, and cancer modelling, as well as stem cell cultures. Future works should consider how synthetic peptide biomaterials can be tuned to match the ECM-niche of various tissues, how they can be translated to the biofabrication of different tissue types and the rheological properties that predict printability. In future, detailed accounts of underlying mechanisms and methods will make it easier for researchers to achieve the progressive pathway of SAPs to SAP-bioinks.

## Data Availability

Data sharing is not applicable to this article as no new data were created or analyzed in this study.
